# Composition and Biological Activity of *Vitis vinifera* Winter Cane Extract on Candida Biofilm

**DOI:** 10.3390/microorganisms9112391

**Published:** 2021-11-19

**Authors:** Zdeněk Kodeš, Maria Vrublevskaya, Markéta Kulišová, Petr Jaroš, Martina Paldrychová, Karolína Pádrová, Kristýna Lokočová, Andrea Palyzová, Olga Maťátková, Irena Kolouchová

**Affiliations:** 1 Department of Biotechnology, University of Chemistry and Technology, 166 28 Prague, Czech Republic; zdenek.kodes@vscht.cz (Z.K.); maria.vrublevskaya@vscht.cz (M.V.); marketa.kulisova@vscht.cz (M.K.); martina.paldrychova@vscht.cz (M.P.); karolina.padrova@vscht.cz (K.P.); olga.matatkova@vscht.cz (O.M.); irena.kolouchova@vscht.cz (I.K.); 2 Department of Biochemistry and Microbiology, University of Chemistry and Technology, 166 28 Prague, Czech Republic; petr.jaros@vscht.cz; 3 Institute of Microbiology, Czech Academy of Sciences, 14220 Prague, Czech Republic; palyzova@biomed.cas.cz

**Keywords:** resveratrol, waste product utilization, prevention of biofilm formation, antioxidant activity

## Abstract

*Vitis vinifera* canes are waste material of grapevine pruning and thus represent cheap source of high-value polyphenols. In view of the fact that resistance of many pathogenic microorganisms to antibiotics is a growing problem, the antimicrobial activity of plant polyphenols is studied as one of the possible approaches. We have investigated the total phenolic content, composition, antioxidant activity, and antifungal activity against *Candida* biofilm of an extract from winter canes and a commercially available extract from blue grapes. Light microscopy and confocal microscopy imaging as well as crystal violet staining were used to quantify and visualize the biofilm. We found a decrease in cell adhesion to the surface depending on the concentration of resveratrol in the cane extract. The biofilm formation was observed as metabolic activity of *Candida albicans*, *Candida parapsilosis* and *Candida krusei* biofilm cells and the minimum biofilm inhibitory concentrations were determined. The highest inhibition of metabolic activity was observed in *Candida albicans* biofilm after treatment with the cane extract (30 mg/L) and blue grape extract (50 mg/L). The composition of cane extract was analyzed and found to be comparatively different from blue grape extract. In addition, the content of total phenolic groups in cane extract was three-times higher (12.75 g_GA_/L). The results showed that cane extract was more effective in preventing biofilm formation than blue grape extract and winter canes have proven to be a potential source of polyphenols for antimicrobial and antibiofilm treatment.

## 1. Introduction

Plant polyphenols represent a group of chemical substances ubiquitously distributed in all higher plants. These secondary metabolites possess free radical scavenging and antimicrobial activity. These properties can be advantageously exploited, especially because of the abundance of polyphenols and their derivatives in various agricultural and food industry waste and by-products and the possibility of convenient extraction by either organic [1] or aqueous solvents [2]. Biosynthesis of polyphenols occurs during plant growth as a response to various biotic and abiotic stresses such as infection, plant injury or UV radiation [3]. Polyphenols are comprised of substances with a wide variety of structures, from simple compounds containing a single hydroxylated aromatic ring to complex polymeric substances. Polyphenols can be characterized based on their structure as non-flavonoid (stilbenes) and flavonoid compounds [4].

Due to their properties, polyphenols can be used in the food industry or medicine in order to avoid oxidative deterioration or undesirable microbial growth [3]. Grapevine (*Vitis vinifera*) is a plant rich in polyphenols and is one of the most commonly grown crops in the world. Therefore, the utilization of winery by-products represents a cheap source of high-quality polyphenolic compounds. The most studied polyphenolic compound is resveratrol. Resveratrol and its derivatives have wide potential in medicinal applications due to its anticancer, antioxidant and antimicrobial effects [5]. Resveratrol belongs to stilbenes, compounds which have a role in the plant response to biotic and abiotic stress, e.g., drought or fungal infection [6]. Resveratrol is found in plants [7] as two geometrical isomers, *E-* (*trans-*) and *Z-* (*cis-*) from which the *trans*- isomer is usually prevalent [8]. Stilbenoid accumulation in *Vitis* ssp. is mostly found in axillary buds, canes, branches and roots. Their concentration varies depending on the part of a plant. For example, Wang et al. [9] found the highest concentration of *trans*-resveratrol in phloem tissue of branches, whereas the lowest concentrations were in leaves [10].

Candidas are clinically relevant human opportunistic pathogens. On the other hand, some strains are also part of natural microflora on the human skin, on the mucous membranes of the digestive tract, the oral cavity or the urogenital system [11]. Nevertheless, candidiasis is one of the most common fungal infections in humans, the most common caused by *Candida albicans*. The pathogenicity of *Candida* is facilitated by several virulence factors, namely, the ability to adhere to medical material or host cells forming highly resistant biofilms. In addition, *Candida* can produce hydrolytic enzymes (phospholipases, proteases and hemolysins) which cause damage to the host cells and enable intrusion into the host tissue. Phospholipases catalyze the hydrolysis of phospholipids to fatty acids. Proteinases facilitate host cell invasion and colonization by cleaving host mucosal proteins and degrading important immunological and structural defense proteins [12]. The therapeutical options for invasive candidiasis are still limited and pose a difficult medical issue. Despite the availability of extended-spectrum triazoles, the incidence of invasive infections and resistance to antifungal therapy continues to increase [13]. The mechanisms of resistance of *Candida* biofilms to fluconazole, but also to the newly used voriconazole, depend on the ongoing phase of biofilm formation. While efflux pumps are used in the early stages of formation, changes in sterol composition are typical of the middle and biofilm maturation phases [14,15]. The fact remains that *Candida* regulates the morphological conversion of the yeast to fibrous form, which along with biofilm formation is an important virulence factor regulated via the QS system [16]. As an example of an anti-pathogenic approach, polyphenolic substances derived from grapevine (*Vitis vinifera*) have been applied in this work, which can disrupt the formation of hyphae and biofilm.

Canes are a waste material produced during grapevine pruning and are usually composted or burned. Therefore, this waste material represents promising and cheap material for further utilization [17]. The canes are known as a rich source of many types of biologically active compounds, such as polyphenols [2]. The potential application of cane extracts is very broad, from complex extracts to individual compounds and has been recently described, e.g., for applications in cosmetics and skin care products [18] or in pharmaceutical or food industries [19]. Their antitumor and anti-inflammatory properties are especially garnering much attention [20]. The potential of bioactive compounds in grapevine extracts as antimicrobial agents has been shown recently [21,22]. Moreira et al. [23] and Oliveira et al. [22] reported the antimicrobial activity of cane and pomace extracts (respectively) on several bacterial strains [24].

In this study, we focused on biological activity of winter cane extract and compared it with commercially accessible blue grape extract. Both *V. vinifera* extracts contain high levels of resveratrol, which has been shown in a number of studies to have antimicrobial, antivirulent, antibiofilm and antifungal activity [25,26]. The content of resveratrol in both extracts was quantified and the amount of extracts used was based on the concentrations of resveratrol contained in them. The biological activity was tested on selected *Candida* species. The problem of antibiotic resistance in many pathogenic microorganisms is one of the most serious complications of treatment. Therefore, new approaches and antimicrobial compounds have been searched for. The combination of antibiotics and bioactive plant extracts can provide synergistic effect which allows lower antibiotics doses.

## 2. Materials and Methods

### 2.1. Yeast and Culture Conditions

Yeast strains, *Candida albicans* DBM 2164 and *Candida parapsilosis* DBM 2165, were obtained from the Collection of the Department of Biochemistry and Microbiology, UCT Prague. *Candida krusei* CCM 8271 was purchased from the Czech Collection of Microorganisms, Masaryk University, Brno. All stock cultures were deposited at –70 °C in 50% glycerol. The cultivation was carried out in yeast extract peptone dextrose (YPD) medium (20 g/L anhydrous D-glucose; 20 g/L pepton; 10 g/L yeast extract) at 30 °C (*Candida albicans* DBM 2164) or 37 °C (*Candida parapsilosis* DBM 2165, *Candida krusei* CCM 8271).

### 2.2. Biologically Active Agents

Pure resveratrol was purchased from Sigma-Aldrich (Burlington, MA, USA). Extract from blue grapes is a commercially available dietary supplement from Interpharma Prague, Czech Republic. Stock solutions were prepared by dissolving in 40% ethanol (EtOH). The composition of *V. vinifera* cane extract and resveratrol quantification is presented in our previous study by Rollova et al. [27].

The extract was prepared by drying winter canes to a constant weight (105 °C), afterwards the canes were crushed and polyphenols were extracted in 40% EtOH (1:4) for 24 h at room temperature in the dark. The efficiency of ethanol extraction was investigated previously, with focus on variable concentrations and temperatures. The concentration of 40% ethanol was chosen as the most effective (both from an economic point of view and in terms of the content of extracted substances) [17,27,28]. The extract was filtered and concentrated 10 times on a vacuum evaporator (40 °C).

For better comparison of blue grape, regarding cane extract’s effects, the numerical value of extract concentration is represented by the concentration of the most abundant stilbene, *trans*-resveratrol. The highest applied concentrations of pure resveratrol (150 mg/L) and blue grape extract (resveratrol content 150 mg/L) and extract from canes (resveratrol content 30 mg/L) were limited by the solubility in 40% ethanol. In all experiments (both planktonic growth and biofilm formation), initial control experiments were performed to determine the effect of EtOH on cell growth, biofilm formation and metabolic activity. It was found that EtOH at concentration 2.5% had no impact. Therefore, all experiments were performed so that this value in culture conditions was not exceeded.

### 2.3. Minimum Inhibitory Concentration Determination

The planktonic growth of *Candida* spp. was examined by monitoring optical density in a microcultivation Bioscreen C device (Oy Growth Curves Ab Ltd., Helsinki, Finland) in a microtiter plate. For inoculum preparation the stock culture stored at −70 °C in 50% glycerol was used. Cultivation was carried out in an Erlenmeyer flask in YPD medium until exponential phase for 24 h, at 30 °C or 37 °C according to the strain. After centrifugation and resuspension in sterile medium, a 30 µL volume of yeast cell suspension (A_600nm_ = 0.1 ± 0.02) was added into each well (final volume 280 µL). Control cells in medium without agents and medium with EtOH (2.5%) were included. Each experiment was performed in three replicates. Minimum concentrations required to inhibit 80% of cell growth (MIC_80_) were determined, according to the definition by Andrews [29], as the lowest concentration that causes at least 80% decrease in growth after a 24 h incubation.

### 2.4. Minimum Biofilm Inhibitory Concentration Determination

The minimum biofilm inhibitory concentration (MBIC_80_) has been determined (procedure modified from Riss et al. [30], as the lowest concentration that causes at least 80% decrease in metabolic activity of biofilm cells after a 24 h cultivation in the presence of biologically active agent. Aliquots of 270 µL of yeast suspension (A_600_ = 0.8 ± 0.02, corresponding to 10^7^ cells per mL in the experiment) (for inoculum cultivation, see Section 2.1), were cultivated in YPD medium in a polystyrene 96-well microtiter plate (TPP AG, Trasadingen, Switzerland) in presence of biologically active substances, for 24 h at 30 °C or 37 °C, on an orbital shaker (150 rpm).

### 2.5. MTT Assay

The metabolic activity of the adherent (biofilm) cells after the treatment of biologically active substances (see Section 2.4) was determined by MTT (3-(4,5-dimethyl-thiazol-2-yl)-2,5-difenyltetrazoliumbromid) assays [30]. MTT was acquired from Sigma-Aldrich (USA). For each experiment, the wells (after biofilm cultivation) were washed three times with physiological saline solution (0.9% NaCl) to remove the planktonic cells. Afterwards, 50 µL MTT solution (1 g/L), 60 µL PBS (phosphate buffered saline with 200 mmol/L glucose) and 15 µL menadione was added into each well. The plate was incubated in the dark at 30 °C or 37 °C (according to the optimal temperature of the tested strain) for 2 h (150 rpm) to form purple formazan crystals. Furthermore, 100 µL of solvent solution (40% dimethylformamide in PBS) was added to enhance dissolving of formazan crystals and the plate was again incubated for 30 min with thorough shaking (230 rpm). A 100 µL aliquot was then transferred into a 96-well microtiter plate, and the color change was determined using a spectrophotometric reader (Tecan, Männedorf, Switzerland) at 570 nm. Experiments were performed with eight parallels in three replicates.

### 2.6. Crystal Violet Staining

Crystal violet (CV) staining was used for total biofilm biomass quantification. The method procedure was modified according to Sabaeifard et al. [31]. The wells were washed three times with saline and 200 μL of 0.1% CV (Carl Roth, Germany) solution was added into each well. Incubation of the plate was carried out for 20 min at room temperature. The wells were then washed three times with saline. CV bound to the biofilm biomass was released by adding 200 μL of 96% ethanol (Penta, Czech Republic). After 10 min incubation at room temperature the colorimetric change was measured using spectrophotometric reader (Tecan, Switzerland) at 580 nm. Experiments were performed with eight parallels in three replicates.

### 2.7. Light Microscopy—Cellavista Device

The area populated by biofilm was visualized in one representative sample using a Cellavista device (Synentech, Elmshorn Germany), as previously described in Kvasnickova et al. [32]. The Cellavista device is an automatic inverted microscope with a high-resolution camera and software for image analysis of the 96-well plates.

### 2.8. Spinning Disc Confocal Microscopy

A confocal microscope with rotating disc equipped with solid state lasers (Olympus, Tokyo, Japan; Andor, Belfast, UK) was used to observed prepared biofilms. Cultivation was carried out under the same condition as described above for MBIC_80_ determination, except for the use of a polystyrene flat bottom microtiter plate (Greiner Bio-One, Gmbh, Frickenhausen, Germany). Control wells and the wells treated with selected concentrations of resveratrol in cane and blue grape extract and pure resveratrol were after cultivation in a microtiter plate stained with the 5 mmol/L SYTO 13 dye (Thermo Fisher Scientific, Waltham, MA, USA) and incubated for 10 min. SYTO 13 binds to the nucleic acids in both live and dead cells and to the extracellular DNA contained in the extracellular matrix of biofilm. To visualize nucleic acids in cells with impaired cell membrane integrity in biofilm, propidium iodide (PI) was used by adding 2 mL of propidium iodide dye (0.02 mg/mL, Sigma-Aldrich, USA) to the cells and incubating for 10 min in the dark.

### 2.9. Determination of Total Antioxidant Activity

The total antioxidant activity of studied extracts was evaluated by the reaction of present antioxidants with a stable free radical DPPH (2,2 Diphenyl-1-picrylhydrazyl) [33]. Studied extracts were diluted in distilled water (1:99) and 100 µL was added into 200 µL of DPPH methanolic solution (5.2 mg/100 mL). When antioxidants are mixed with DPPH solution, the color of solution is turned to yellow from purple. After incubation for 15 min at room temperature in the dark, the color change was measured using a spectrophotometric reader (Tecan, Switzerland) at 517 nm. As blank sample, 100 µL of distilled water with 200 µL of DPPH solution was used. Gallic acid was used as a standard (2.5–20 mg/L). Antioxidative activity was expressed as gallic acid equivalent concentration (g_GA_/mL).

### 2.10. The Amount of Total Phenolic Groups

The amount of total phenolic groups was determined using Folin–Ciocalteu reagent procedure [34]. Furthermore, 165 µL of Folin–Ciocalteu reagent was mixed with 15 µL of the extract diluted in distilled water (1:99). After 3 min, 60 µL 2M Na_2_CO_3_ and 80 µL of distilled water were added and incubated for 60 min in dark. Finally, the absorbance was measured at 700 nm. Gallic acid was used as a standard (30–100 mg/L) and the amount of total phenolic groups was expressed as gallic acid equivalent concentration (g_GA_/mL).

### 2.11. Statistical Analysis

Dixon’s Q test was performed to detect outliers in data acquired by the crystal violet staining and the MTT assay. Arithmetic means and standard deviations were calculated from the data for each concentration tested in each experiment. The significance of the difference between control and effective concentration of biologically active agent was determined by one-way analysis of variance (ANOVA) with significance of *p* < 0.05.

## 3. Results and Discussion

Understanding of chemical composition and potential biological properties of plant extracts is crucial for the understanding of their properties and their further use. The antimicrobial, antivirulent, antibiofilm and antifungal activity of resveratrol is still the subject of a number of studies [25,26]. The inhibitory effect of resveratrol on the bacterial biofilm has been described, for example, by Coenye et al. [35], Lee et al. [36] and Augustine et al. [25]. In a study by Sheng et al. [37], the ability of resveratrol to reduce the expression of the lasI, lasR, rhlI and rhlR genes and to disrupt pyocyanin production in *P. aeruginosa* PAO1 was reported. Resveratrol was also identified as a potential inducer of *C. albicans* cell apoptosis [26].

Both *V. vinifera* extracts contain high levels of resveratrol and both have shown high antifungal activity. The more pronounced inhibitory action of the extracts compared to pure resveratrol is probably due to the synergistic interactions and effects of several substances. Some flavonoids are known to increase the bioavailability of co-administered substances, which is attributed to their inhibitory effect on enzymes that play an important role in metabolism [38]. The synergistic action of several polyphenols at once was the explanation for the more pronounced antibacterial action of *Vitis rotundifolia* extracts (in comparison with pure quercetin and catechin) in the study of Xu et al. [39]. Bacterial growth inhibition by *trans*-ε-viniferin isolated from leaves of *Vitis amurensis* (also contained in annuals extract) has also been reported in bacteria [40].

We focused on extracts from winter canes, by-products of *V. vinifera* cultivation in winemaking industry. UHPLC-HRMS/MS analysis indicated the total phenolic composition of the cane extract. Results of the investigation are presented in Rollova et al. [27]. Among detected substances were those with known biological and antioxidative activity. A significant group in the cane extract was stilbenes. Among those detected were resveratrol and its derivatives viniferol A, ε-viniferin, angolensin and copalliferol B. Their antimicrobial character was described by many studies against various microorganisms [3,41,42]. Another group found were flavonoids—procyanidin and quercetin glucuronide. Rhimi et al. [43] reported antibacterial and antifungal activities against *Candida* spp. of several flavonoids, including quercetin glucuronide. Procyanidin exposition proved an ability to inhibit bacterial growth and biofilm formation [44]. An interesting constituent of the extract was chrysophanic acid-9-anthrone, whose antifungal activity was observed against *Candida albicans*, *Saccharomyces cerevisiae* and *Aspergillus niger* [45].

Biological activity of cane extract was compared with commercially available blue grape extract. Total phenolic content was described by Paldrychova et al. [28]. From the analysis it is obvious that the composition of both extracts is comparatively different. Such a difference may have significant effect on further application. *C. albicans*, *C. parapsilosis* and *C. krusei* were treated to show the influence of extract composition on planktonic cell growth and biofilm formation. Table 1 summarizes MIC_80_ values of planktonic growing cells after exposition to studied extracts and pure resveratrol. Due to limited solubility in 40% ethanol, higher concentrations of cane extract (30 mg/L), blue grape extract (150 mg/L) and pure resveratrol (150 mg/L) could not be applied.

Values of MIC_80_ summarized in Table 1 confirm that the biological activity of studied extracts is dependent not only on the composition, but also on the type of microorganism. Nevertheless, cane extract was found to be more effective than blue grape extract or pure resveratrol with lowest MIC_80_ against *C. albicans* (5 mg/L) and *C. parapsilosis* (30 mg/L). Blue grape extract had similar resulting effect as pure resveratrol. MIC_80_ was achieved only in the case of *C. albicans* (80 mg/L) after resveratrol treatment. El Darra et al. [46] analyzed the composition of different grapevine varieties and examined antimicrobial activities of obtained extracts. Analyses showed a great variability in the composition of phenolic compounds. In addition, antimicrobial tests demonstrated different reaction of treated microorganism depending on the phenolic composition. Similar results were reported by Baydar et al. [47], who compared phenolic content in grape seeds and bagasse of *Vitis vinifera* L. and the effect of their extracts on several pathogenic bacteria.

Nowadays, there are few studies focused on properties and effects of cane extract. This waste product of *V. vinifera* pruning still represents unexploited agronomical waste material, due to its high content of biologically active compounds. Schnee et al. [48] reported that the cane extract represents a promising antibacterial agent. However, the composition of their extract was significantly different from the composition of cane extract reported in our study. From analyzed stilbenoids, both extracts contained only resveratrol and ε-viniferin. Winter cane extract proved to also be efficient as an antibiofilm agent. The metabolic activity of *Candida* strains cells studied was measured during the biofilm formation and the minimum biofilm inhibitory concentration (MBIC_80_) was determined by the MTT assay. MBIC_80_ of cane extract was 30 mg/L for *C. albicans* and *C. krusei* (Table 2), making it more effective compared to blue grape extract or pure resveratrol. Based on these results, it is probable that the antimicrobial behavior of the extracts will be greatly influenced by the representation of flavonoid compounds.

The investigation of total phenolic content determined by Folin–Ciocalteu method showed a significant difference between studied extracts. The content of total phenolic groups in cane extract was three-times higher (12.75 g_GA_/L) than blue grape extract (4.08 g_GA_/L). That difference corresponded with the efficiency of antifungal activity of the studied extracts, which is described above. The same dependence was reported by Katalinic et al. [49], the extract with the highest phenolic content provided the best antibacterial activity. By contrast, the difference in antioxidant activity of both extracts was not statistically significant; for cane extract 2.17 g_GA_/L and 1.92 g_GA_/L for blue grape extract, respectively. That was also observed by Katalinic et al. [49], who concluded that the total antioxidant capacity was probably the result of synergistic activity of all phenolic constituents, not dependent on single phenolic content. Nevertheless, the total phenolic compounds composition and their quantity seem to be crucial for the biological activity.

**Figure 1:**
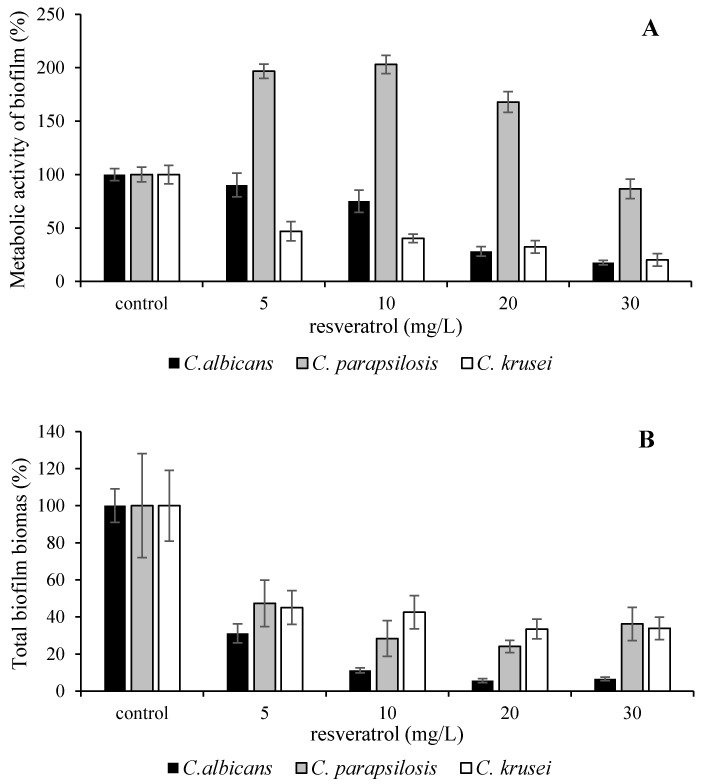
The influence of resveratrol in cane extract on *C. albicans* DBM 2164, *C. parapsilosis* DBM 2165 and *C. krusei* CCM 8271. The metabolic activity of biofilm (**A**); the total biofilm biomass (**B**); control 100% (no agent). Error bars represent standard deviation.

**Figure 2:**
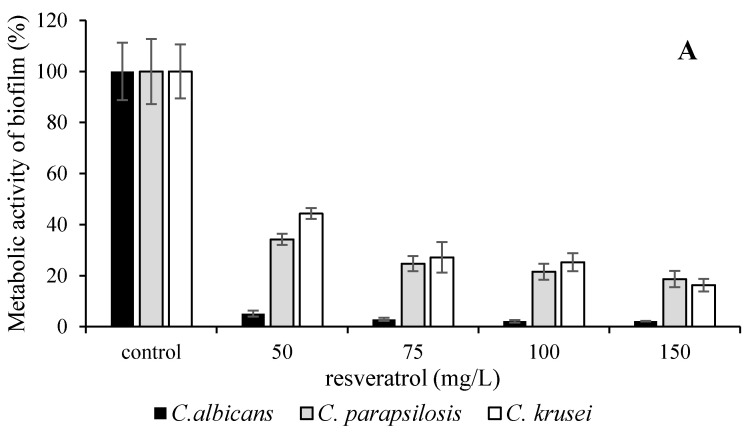

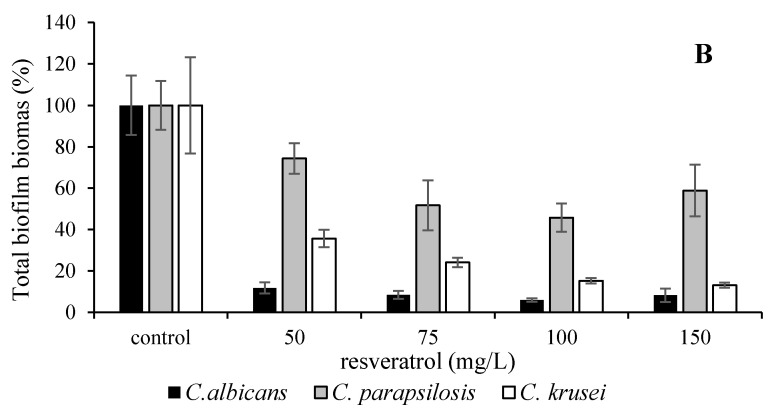
The influence of resveratrol in blue grape extract on *C. albicans* DBM 2164, *C. parapsilosis* DBM 2165 and *C. krusei* CCM 8271. The metabolic activity of biofilm (**A**); the total biofilm biomass (**B**); control 100% (no agent). Error bars represent standard deviation.

Figure 1 and Figure 2 illustrate how the metabolic activity and total biomass content were influenced under studied conditions. The highest inhibition of metabolic activity of biofilm was observed in *C. albicans*, significant inhibition confirmed by the one-way ANOVA test (significant difference at *p* < 0.05) was observed at concentrations higher than 20 mg/L. The exposition of yeasts to both extracts led to decrease in metabolic activity with the exception of *C. parapsilosis*. The treatment of *C. parapsilosis* by cane extract caused decrease in total biomass under all used concentrations (Figure 1B), but the metabolic activity was significantly enhanced, by 100%. That response was observed after treatment of *C. parapsilosis* by resveratrol concentrations of 5–20 mg/L in cane extract. Based on our results, we assume that yeast responses to cane extracts are concentration-dependent. At high concentrations, biologically active substances exhibit antimicrobial activities on susceptible cells, while subinhibitory concentrations induce diverse biological responses in yeasts, including in some cases, a stress-related defensive reactions such as defensive growth of biofilm (Figure 1A). Similar results to these findings are reported by Imbert et al. [50].

Blue grape extract also had inhibitive effect on biofilm formation but only in higher concentrations (Figure 2). The best response was observed in the treatment of *C. albicans*, MBIC_80_ was determined to be 50 mg/L by one-way ANOVA test (significant difference at *p* < 0.05). Pure resveratrol was shown to be the weakest agent without significant effect. The total biofilm biomass determination (by crystal violet staining) was supplemented with optical microscopic visualization of the biofilm formation.

**Figure 3:**
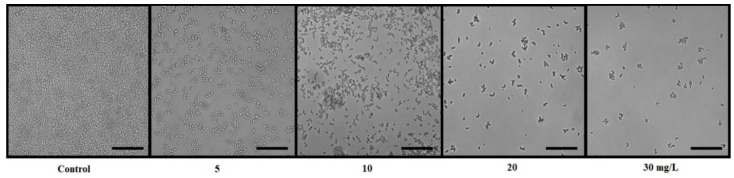
The influence of resveratrol in cane extract on *C. albicans* DBM 2164 biofilm formation visualized by a Cellavista device; scale bar 400 μm.

Figure 3 illustrates the example of *C. albicans* treatment by increasing concentration of resveratrol in cane extract. The pictures show a decrease in cell adhesion to the surface depending on the concentration. Changes in morphology (hyphae formation) in the genus *Candida* may indicate a change in their pathogenicity as hyphae-like cells are considered a virulence factor. As can be seen from Figure 3, there was no change in morphology observed in our study, so it can be assumed that there was no manifestation of this virulence factor causing the pathogenicity of the strains.

**Figure 4:**
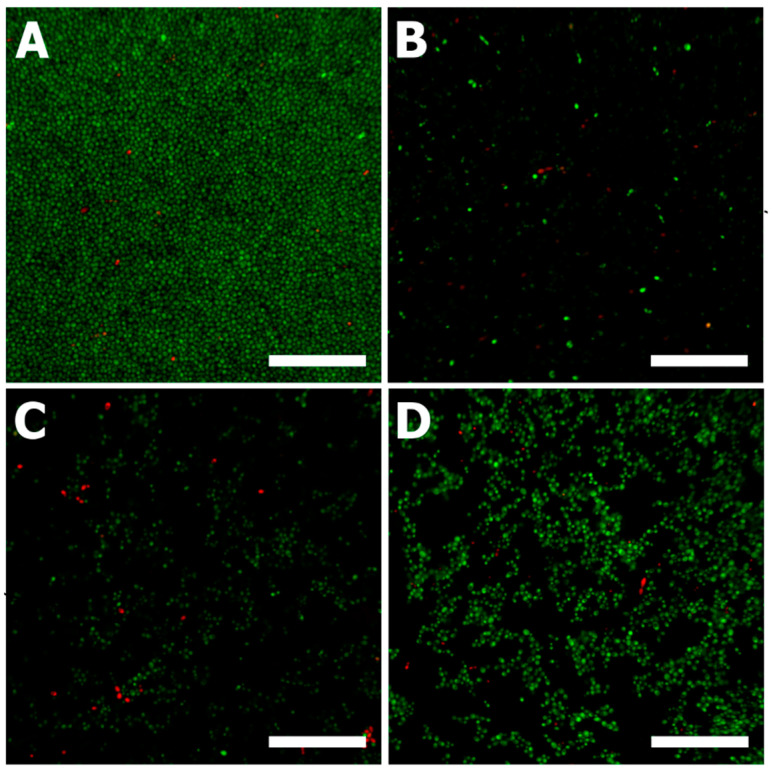
Imaging of *C. albicans* DBM 2164 biofilm by confocal microscopy with rotating discs. Control (**A**), 100 mg/L of resveratrol in blue grape extract (**B**), 10 mg/L of resveratrol in cane extract (**C**), 100 mg/L pure resveratrol (**D**); scale bar 400 μm.

To confirm the effect of extracts on biofilm formation, confocal microscopy was used (Figure 4) and the visualization of live (green) and dead (red) cells was performed. The treatment of *C. albicans* adherent cells proved the significant difference in effectiveness of applied substances, especially of the grapevine extracts (Figure 4B,C) in comparison with control and pure resveratrol (Figure 4A,D, respectively), all in concord with results described above.

Our results may be potentially useful in finding a solution to one of the major world health problems, which is the emergence of multi-resistant microorganisms to conventional drugs [51]. Especially in the emergence of multi-resistant strains of *Candida,* both albicans and non-albicans species (such as *Candida auris* or *Candida glabrata*). Among possible treatments for these infections are use of biologically active substances from natural resources, such as peptides and polypeptides (e.g., crotamine, antimicrobial polypeptide from the South American rattlesnake *Crotalus durissus terrificus*, with strong efficiency against multi-resistant clinical isolates [52]). Another group of promising substances are synthetic surfactants based on amino acids, which affect the cell membrane permeability as well as the mitochondria to target even multi-resistant strains and even act synergistically with antifungals [53]. Or we can propose another possibility in the application of plant extracts with their antifungal and antibiofilm effects. These can be based on our results where we have proved these effects on two *Candida* species. Even more beneficial in the goal of effective control would be their combination in combination therapy exploring synergism antimycotics [54,55]. Among the most studied plants with antifungal effects against multidrug-resistant species are representatives of Apiacae, Asteraceae, Fabaceae and Myrtaceae [56]. Plant extracts’ effectiveness is determined by their composition and is often credited to the high content of substances with antioxidant properties such as phenolic substances [57] and antifungals (e.g., *Prockia crusis*) [58].

## 4. Conclusions

We have investigated the effectivity of extracts from *Vitis vinifera* canes against *Candida albicans*, *Candida parapsilosis* and *Candida krusei* biofilm. Biofilm formation inhibition was confirmed and the minimum biofilm inhibitory concentrations were found. The composition of cane extract was analyzed and their total phenolic content, composition and antioxidant activity determined. The results showed that *Vitis vinifera* cane extract was more effective in inhibiting biofilm formation than pure resveratrol and therefore synergistic activity is supposed in the antimicrobial and antibiofilm treatment, however, despite the obvious potential of these biologically active waste product derivatives, more research is needed.

## Figures and Tables

**Table 1:** Minimum inhibitory concentrations (MIC_80_) of blue grape extract, cane extract and resveratrol for *C. albicans* DBM 2164, *C. parapsilosis* DBM 2165 and *C. krusei* CCM 8271.

		MIC_80_ (mg/L)
*C. albicans* DBM 2164	Cane extract	5
	Blue grape extract	>150
	Resveratrol	80
*C. parapsilosis* DBM 2165	Cane extract	30
	Blue grape extract	>150
	Resveratrol	>150
*C. krusei* CCM 8271	Cane extract	>30
	Blue grape extract	>150
	Resveratrol	>150

**Table 2:** Minimum biofilm inhibitory concentrations (MBIC_80_) of blue grape extract, cane extract and resveratrol for *C. albicans* DBM 2164, *C. parapsilosis* DBM 2165 and *C. krusei* CCM 8271.

		MBIC_80_ (mg/L)
*C. albicans* DBM 2164	Cane extract	30
	Blue grape extract	50
	Resveratrol	>150
*C. parapsilosis* DBM 2165	Cane extract	>30
	Blue grape extract	150
	Resveratrol	150
*C. krusei* CCM 8271	Cane extract	30
	Blue grape extract	100
	Resveratrol	>150

## Data Availability

Not applicable.
